# Association of *MTHFR* C677T polymorphism and type 2 diabetes mellitus (T2DM) susceptibility

**DOI:** 10.1002/mgg3.1020

**Published:** 2019-10-30

**Authors:** Yanzi Meng, Xiaoling Liu, Kai Ma, Lili Zhang, Mao Lu, Minsu Zhao, Min‐Xin Guan, Guijun Qin

**Affiliations:** ^1^ Department of Endocrinology and Metabolism The First Affiliated Hospital of Zhengzhou University Zhengzhou Henan China; ^2^ Jincheng General Hospital Jincheng Coal General Hospital Jincheng Shannxi China; ^3^ Endocrinology Department Affiliated Hospital of Guilin Medical University Guilin Guangxi China; ^4^ Hematology Department Jincheng People’s Hospital Jincheng Shannxi China; ^5^ Endocrinology Department Jincheng General Hospital Jincheng Coal General Hospital Jincheng Shannxi China; ^6^ Laboratory Medicine Department Jincheng General Hospital Jincheng Coal General Hospital Jincheng Shannxi China; ^7^ Endocrinology Department Jincheng People’s Hospital Jincheng Shannxi China; ^8^ Institute of Genetics Zhejiang University Hangzhou Zhejiang China

**Keywords:** C677T, meta‐analysis, *MTHFR*, polymorphism, T2DM

## Abstract

**Introduction:**

Methylenetetrahydrofolate reductase (MTHFR) is essential in mediating folate metabolism, and thus plays an important role in diabetes and diabetic complications. *MTHFR* C677T (rs1801133 C>T) polymorphism has been proposed to be linked with type 2 diabetes mellitus (T2DM) susceptibility. However, the conclusions are inconsistent. Therefore, we rechecked their linkage aiming to obtain a more reliable estimation by performing an updated meta‐analysis.

**Methods:**

We searched electronic databases PubMed, EMBASE, CNKI, and Wanfang to obtain studies updated to October 2019.

**Results:**

After carefully screening, we finally incorporated 68 studies with 10,812 cases and 8,745 controls. The genotype frequency of C677T polymorphism was analyzed pooled to generate odds ratios (ORs) and 95% confidence intervals (CIs). Pooled results presented that *MTHFR* C677T polymorphism was significantly associated with T2DM under homozygous (OR = 1.64, 95% CI = 1.39–1.94), heterozygous (OR = 1.38, 95% CI = 1.20–1.59), recessive (OR = 1.41, 95% CI = 1.23–1.61), dominant (OR = 1.47, 95% CI = 1.27–1.70), and allele (OR = 1.37, 95% CI = 1.23–1.52) genetic models. Stratified analysis demonstrated that C677T genotype was associated with T2DM in Asian populations, but not Caucasian and African populations.

**Conclusion:**

Our results indicated that *MTHFR* C677T polymorphism confers to T2DM, especially in Asian populations. Much more large‐scale case–control studies are needed to strengthen such conclusion in the future.

## Graphical abstract

Pooled results presented that *MTHFR* C677T polymorphism was significantly associated with type 2 diabetes mellitus (T2DM) under homozygous (odds ratio [OR] = 1.61, 95% confidence interval [CI] = 1.37–1.90), heterozygous (OR = 1.32, 95% CI = 1.15–1.51), recessive (OR = 1.39, 95% CI = 1.22–1.58), dominant (OR = 1.40, 95% CI = 1.22–1.60), and allele (OR = 1.32, 95% CI = 1.20–1.45) genetic models. Stratified analysis demonstrated that C677T genotype was associated with T2DM in Asian populations, but not Caucasian and African populations. Our results indicated that *MTHFR* C677T polymorphism confers to T2DM, especially in Asian populations.

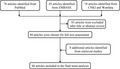

## 1 INTRODUCTION

Type 2 diabetes mellitus (T2DM) is a major public health problem that not only affects individual life quality, but also increases social economic burden (DeFronzo et al., 2015). The frequency of T2DM in China is increasing quickly, with estimation of about 380 million T2DM patients by 2025 (van Dieren, Beulens, van der Schouw, Grobbee, & Neal, 2010). The etiology of T2DM remains partly elucidated. Evidences suggest that T2DM is a complex disease caused by the combinations of environmental and genetic risk factors (Wareham, Franks, & Harding, 2002; Zeggini et al., 2007).

Previous reports showed that individuals with insufficient intake of folic acid were more likely to have T2DM. Folate is a methyl group donor in the synthesis of intracellular methylation reactions and de novo deoxynucleoside (Blount et al., 1997). When folate deficiency, the DNA stability will be impaired (Duthie, 1999). Methylenetetrahydrofolate reductase (MTHFR) is a folate‐metabolizing enzyme that participates in folic acid circulation and DNA synthesis (Friso et al., 2002). MTHFR catalyzes the irreversible reduction of 5,10‐methylenetetrahydrofolate to 5‐methyltetrahydrofolate (Niclot et al., 2006). Dysfunction or low activity of MTHFR may decrease the level of methyl pool; consequently, it inhibits the successful deoxynucleoside synthesis and intracellular methylation reactions (Rozen, 1997).

The human gene *MTHFR* (OMIM number: 607093) is located on chromosome 1p36.3. Of all the identified SNPs, C677T (Ala222Val, rs1801133 C>T) is one of the most investigated genetic variations (Adinolfi et al., 2005; Liew & Gupta, 2015). The C677T polymorphism is a C to T transition at base pair 677, which results in the amino acid transition from Ala to Val. Such amino acid transition significantly decreases the activity of MTHFR (Weisberg, Tran, Christensen, Sibani, & Rozen, 1998). Recent data suggested that there exist an association between C677T and the susceptibility of T2DM. However, the role of C667T in risk of T2DM was discrepant. Several meta‐analyses that were conducted to solve this conflicting role somehow failed. To get a precise estimation, we re‐analyzed the role of *MTHFR* C677T on T2DM via including larger eligible investigations.

## 2 MATERIALS AND METHODS

### 2.1 Literature search

We carried out a comprehensive literature search in the following databases: PubMed, EMBASE, CNKI, and Wanfang. The searching was updated to October 2019 without any language limitations. The combination of the following search terms was adopted: ‘*MTHFR* or methylenetetrahydrofolate reductase’, and ‘polymorphism or polymorphisms or SNP or single nucleotide polymorphism or variant’ and ‘diabetes or mellitus or diabetes mellitus or T2DM’. To expand the included studies, we also retrieved eligible references from the selected studies. The GenBank reference sequence and version number for the gene is: *MTHFR* (NM_005957.5).

### 2.2 Inclusion/exclusion criteria

We set the following criteria when performing the selection work: (a) evaluating the association of *MTHFR* C677T polymorphism with T2DM risk; (b) case–control design; (c) odds ratios (ORs) and their 95% confidence intervals (CIs) were able to obtain; and (d) reports Hardy–Weinberg equilibrium (HWE). Exclusion criteria were as follows: (a) reviews or meta‐analyses; (b) case‐only studies or case reports; and (c) duplicate publications.

### 2.3 Data extraction

We arranged three authors to handle data extraction: two authors to extract data independently and one author to resolve the disagreement. The following data were selectively extracted from each study: first author's surname, year of publication, country, ethnicity, genotyping methods, and genotypic distribution. The stratification analysis was conducted by ethnicity (Asians, Caucasians, and Africans) and HWE (HWE <0.05 and HWE >0.05).

### 2.4 Statistical methods

STATA 11.0 software (Stata Corporation) was adopted to conduct the current meta‐analysis. We first used Chi‐square test to check whether the genotype frequency of C677T among the controls was in HWE. After that, we determined the relationship between *MTHFR* C677T polymorphism and T2DM risk by calculating pooled ORs with the corresponding 95% CIs. We totally used five genetic models: homozygote model (TT vs. CC), heterozygote model (CT vs. CC), recessive model (TT vs. CT/CC), dominant model (CT/TT vs. CC), and allele model (T vs. C) to detect such relationship. Stratification analyses were also taken by ethnicity (Asian, Caucasian, and African) and HWE (HWE <0.05 and HWE >0.05), aiming to detect the source of heterogeneity. We carried out Chi‐square‐based Q statistic test and inconsistency index statistics (*I* ^2^) to calculate heterogeneity between study results. If the studies were homogeneous (with *p^het^* < .10 or *I* ^2^ > 50%), the random‐effects model (the DerSimonian and Laird method) was chosen. Otherwise, ORs were calculated using the fixed‐effects model (the Mantel‐Haenszel method). Sensitivity analysis was performed to assess the strength of the conclusion, by sequentially excluding each study at a time. Begg's funnel plot and Egger's linear regression test were conducted to assess publication bias. We also conducted quality assessment to detect the quality of each study using the quality assessment criteria (He et al., 2014). All the statistics were two‐sided. *p* < .05 was considered as significant.

## 3 RESULTS

### 3.1 Study search

**Figure 1:**
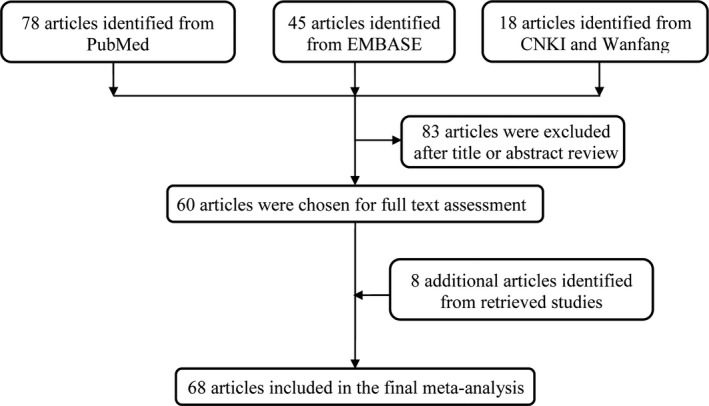
Search flow diagram

General process of publication selection was graphically shown in Figure 1. Initial retrieval from PubMed and EMBASE databases got a total of 78 and 45 potentially relevant published records, respectively. We also obtained 18 articles from Chinese databases CNKI and Wanfang. After titles and abstracts screening, 81 nonrelevant records were excluded. The remaining 60 articles and eight additional articles identified from retrieved studies were included in the final meta‐analysis (Al‐Harbi et al., 2015; Al‐Salihi, Ajeena, Al‐Kashwan, & Al‐Lebban, 2016; Zidan, El Mougy, Moustafa, El attar, & Mohamed, 2019; Benrahma et al., 2012; Bluthner et al., 1999; Cao, Huang, Mao, & Gao, 2005; Chang et al., 2011; Chen, Ning, Zhu, Li, & Shi, 2004; Chen et al., 2010; P. Chen, Pan, Sun, Bai, & Fu, 2008; Dai & Yu, 2012; El Hajj Chehadeh et al., 2016; Eroglu et al., 2007; Errera et al., 2006; Fekih‐Mrissa et al., 2017; Fujita et al., 1999; Guo, Pan, Chu, Guo, & Sun, 2005; Guo et al., 2002; Hu, Zhang, Fang, Qin, & Liu, 2009; Hu, Gan, Li, & Bi, 2001; Jimenez‐Ramirez et al., 2017; Ksiazek, Bednarek‐Skublewska, & Buraczynska, 2004; Lin, Wang, & Liu, 2009; Liu et al., 2014; Luo, Yan, Li, Cheng, & Song, 2007; Luo, Yan, Ma, Cheng, & Song, 2008; Mao, Gao, Qin, & Shi, 2004; Mehri et al., 2010; Mei, Chen, & Zheng, 2012; Mtiraoui et al., 2007; Neugebauer, Baba, & Watanabe, 1998; Nithya et al., 2017; Odawara & Yamashita, 1999; Pirozzi et al., 2018; Qiu, 2009; Rahimi et al., 2009; Ramanathan, Harichandana, Kannan, Elumalai, & Sfd, 2019; Raza, Abbas, Siddiqi, & Mahdi, 2017; Settin, El‐Baz, Ismaeel, Tolba, & Allah, 2015; Shang, Wang, & Liu, 2017; Shi, He, Cheng, Wang, & Liu, 2006; J. Shi, Li, Yu, Chen, & Tao, 2002; Shpichinetsky et al., 2000; Soares et al., 2008; J. Sun, Xu, Xue, Zhu, & Lu, 2005; Sun, Xu, & Zhu, 2001; Sun, Xu, Zhu, & Lu, 2004; Sun, Xu, Lu, & Zhu, 2009; Sun, Chen, et al., 2004; Sun, Wang, Shi, & Yang, 2013; Wang et al., 2017; Wang, Hu, Xiao, & Wan, 2014; Wang, Wang, & Li, 2018; Wang, Wang, Xue, Chen, & Zou, 2001; Wang, Wang, Xue, Cheng, et al., 2001; Wen, Lu, Li, Wu, & Zhang, 2008; Wirta et al., 1998; Xiao, Hu, Shan, Guan, & Ren, 2006; Xu, Zhang, Shan, & Ma, 2003; Yang, Lu, & Pan, 2001; Yilmaz, Agachan, Ergen, Karaalib, & Isbir, 2004; Yoshioka et al., 2004; Yue, Liu, Kang, Hu, & Qiu, 2006; Zhang, Li, Liu, & Hu, 2007; Zhang, Xiang, Weng, & Li, 2002; Zhang & Liu, 2009; Zhi et al., 2016; Zhou, Li, & Zhang, 2004).

### 3.2 Study characteristics

The study characteristics of the final selected studies were presented in Table 1. A total of 68 studies with 10,812 cases and 8,745 controls were included in our final meta‐analysis. Among these eligible studies, 52 were done on Asians, 11 studies were done on Caucasians, and five studies were done on Africans. As to the HWE, genotype distribution in the controls of 52 studies was agreed with the HWE, and 16 studies were not. We also classified the studies into low‐quality studies (48 studies) and high‐quality studies (20 studies) by quality score.

**Table 1:** Characteristics of studies included in the current meta‐analysis

Surname	Year	Country	Ethnicity	Genotype method	Case				Control				HWE	Score
					CC	CT	TT	Total	CC	CT	TT	Total		
Neugebauer	1998	Japan	Asian	PCR‐RFLP	24	31	12	67	86	43	17	146	0.003	6
Wirta V	1998	Finland	Caucasian	PCR‐RFLP	46	30	8	84	60	48	7	115	0.520	8
Bluthner	1999	Germany/Poland	Caucasian	PCR‐RFLP	74	50	23	147	67	68	15	150	0.708	6
Fujita	1999	Japan	Asian	PCR‐RFLP	31	57	17	105	20	39	9	68	0.142	7
Odawara	1999	Japan	Asian	PCR‐RFLP	52	65	26	143	38	68	25	131	0.578	7
Shpichinetsky	2000	Israel	Caucasian	PCR‐RFLP	23	22	10	55	21	16	6	43	0.316	3
Hu S	2001	China	Asian	PCR‐RFLP	49	48	16	113	30	24	1	55	0.121	5
Sun J	2001	China	Asian	PCR‐RFLP	32	33	20	85	10	16	31	57	0.008	4
Wang L	2001	China	Asian	PCR‐RFLP	52	68	41	161	37	36	12	85	0.502	5
Wang L	2001	China	Asian	PCR‐RFLP	65	75	39	179	37	38	10	85	0.959	7
Yang G	2001	China	Asian	PCR‐RFLP	17	27	23	67	26	28	8	62	0.914	6
Guo Q	2002	China	Asian	PCR‐RFLP	12	19	22	53	12	11	5	28	0.391	7
Shi J	2002	China	Asian	PCR‐RFLP	12	31	7	50	22	29	5	56	0.291	5
Zhang G	2002	China	Asian	PCR‐RFLP	56	108	34	198	40	49	11	100	0.484	7
Xu J	2003	China	Asian	PCR‐RFLP	39	54	30	123	20	25	7	52	0.853	8
Chen A	2004	China	Asian	PCR‐RFLP	24	45	22	91	21	9	5	35	0.038	7
Ksiazek P	2004	Poland	Caucasian	PCR‐RFLP	159	123	44	326	71	83	16	170	0.237	10
Mao L	2004	China	Asian	PCR‐RFLP	35	37	11	83	26	18	3	47	0.960	8
Sun J	2004	China	Asian	PCR‐RFLP	102	76	42	220	74	34	22	130	<0.001	9
Sun L	2004	China	Asian	PCR‐RFLP	27	52	27	106	29	18	3	50	0.925	7
Yilmaz H	2004	Turkey	Caucasian	PCR‐RFLP	121	98	30	249	101	93	20	214	0.831	8
Yoshioka K	2004	Japan	Asian	PCR‐RFLP	21	13	6	40	71	107	29	207	0.260	9
Zhou J	2004	China	Asian	PCR‐RFLP	16	78	45	139	8	31	30	69	0.998	8
Cao H	2005	China	Asian	PCR‐RFLP	14	20	6	40	26	18	3	47	0.960	7
Guo L	2005	China	Asian	PCR‐RFLP	60	51	50	161	58	34	35	127	<0.001	8
Sun J	2005	China	Asian	PCR‐RFLP	101	78	49	228	63	31	20	114	<0.001	10
Errera FI	2006	Brazil	Caucasian	PCR‐RFLP	44	41	10	95	36	57	14	107	0.244	9
Shi C	2006	China	Asian	PCR‐RFLP	108	60	18	186	68	34	7	109	0.338	8
Xiao Y	2006	China	Asian	PCR‐RFLP	16	53	4	73	47	25	1	73	0.245	7
Yue H	2006	China	Asian	PCR‐RFLP	66	131	55	252	17	11	2	30	0.903	8
Eroglu Z	2007	Turkey	Caucasian	PCR‐RFLP	51	45	7	103	63	58	7	128	0.171	7
Luo D	2007	China	Asian	PCR‐RFLP	65	102	26	193	42	35	14	91	0.151	7
Mtiraoui N	2007	Tunisia	Caucasian	PCR‐RFLP	163	135	62	360	270	94	36	400	<0.001	12
Zhang C	2007	China	Asian	PCR‐RFLP	28	29	19	76	34	19	12	65	0.006	8
Chen P	2008	China	Asian	PCR‐RFLP	19	70	27	116	14	73	37	124	0.014	9
Luo D	2008	China	Asian	PCR‐RFLP	59	63	19	141	43	31	11	85	0.166	8
Soares AL	2008	Brazil	Caucasian	PCR‐RFLP	15	8	2	25	9	5	2	16	0.363	3
Wen J	2008	China	Asian	PCR‐RFLP	22	50	23	95	27	25	5	57	0.816	6
Hu L	2009	China	Asian	PCR‐RFLP	47	63	49	159	26	17	9	52	0.053	7
Lin R	2009	China	Asian	PCR‐RFLP	56	36	47	139	93	22	24	139	<0.001	10
Qiu Y	2009	China	Asian	PCR‐RFLP	83	68	48	199	53	29	18	100	<0.001	9
Rahimi Z	2009	Iran	Asian	PCR‐RFLP	33	27	5	65	33	22	4	59	0.898	5
Sun J	2009	China	Asian	PCR‐RFLP	94	73	48	215	78	38	26	142	<0.001	10
Zhang Q	2009	China	Asian	PCR‐RFLP	66	94	66	226	26	17	9	52	0.053	8
Chen A	2010	China	Asian	PCR‐RFLP	57	74	27	158	34	17	4	55	0.373	8
Mehri S	2010	Tunisia	African	PCR‐RFLP	50	49	16	115	66	38	12	116	0.078	8
Chang YH	2011	China	Asian	PCR	1	25	30	56	3	23	36	62	0.781	6
Houda Benrahma	2012	Morocco	African	PCR‐RFLP	160	97	25	282	114	122	26	262	0.420	10
Dai H	2012	China	Asian	PCR‐RFLP	51	54	15	120	31	27	2	60	0.176	8
Mei Q	2012	China	Asian	PCR‐RFLP	17	51	23	91	17	70	37	124	0.076	8
Sun L	2013	China	Asian	PCR‐RFLP	180	243	48	471	30	42	6	78	0.094	11
Liu K	2014	China	Asian	PCR‐RFLP	103	54	6	163	54	23	0	77	0.123	8
Han Wang	2014	China	Asian	TaqMan	234	293	66	593	298	312	70	680	0.377	12
Al‐Harbi EM	2015	Bahrain	Asian	PCR‐RFLP	116	43	12	171	135	47	6	188	0.449	10
Ahmad Settin	2015	Egypt	African	PCR‐RFLP	111	65	27	203	156	135	20	311	0.195	11
Al‐Salihi NJ	2016	Iraqi	Asian	PCR‐RFLP	28	28	5	61	12	10	0	22	0.167	4
El Hajj Chehadeh SW	2016	United Arab Emirates	Asian	TaqMan	155	49	5	209	132	27	10	169	<0.001	10
Xueyuan Zhi	2016	China	Asian	TaqMan	28	86	66	180	76	172	102	350	0.826	11
Fekih‐Mrissa N	2017	Tunisia	African	PCR‐RFLP	56	102	2	160	124	68	8	200	0.726	11
Jimenez‐Ramirez FJ	2017	Puerto Rico	Caucasian	PCR‐RFLP	72	8	9	89	184	159	57	400	0.020	10
K Nithya	2017	India	Asian	PCR‐RFLP	173	25	2	200	94	6	0	100	0.757	10
Raza ST	2017	India	Asian	PCR‐RFLP	152	162	65	379	102	52	26	180	<0.001	11
Shang G	2017	China	Asian	PCR‐RFLP	84	106	36	226	66	91	37	194	0.573	11
Wang D	2017	China	Asian	PCR‐RFLP	69	72	21	162	162	127	13	302	0.052	10
Pirozzi FF	2018	Brazil	Caucasian	PCR‐RFLP	17	22	8	47	30	38	9	77	0.560	7
Wang J	2018	China	Asian	PCR‐RFLP	176	101	103	380	183	70	53	306	<0.001	11
Ramanathan G	2019	India	Asian	PCR‐RFLP	72	71	2	145	81	19	0	100	0.293	10
Zidan	2019	Egypt	African	PCR‐RFLP	30	51	39	120	54	6	0	60	0.683	9

Abbreviations: HWE, Hardy–Weinberg equilibrium; PCR‐RFLP, polymerase chain reaction‐restriction fragment length polymorphism.

### 3.3 Meta‐analysis results

**Figure 2:**
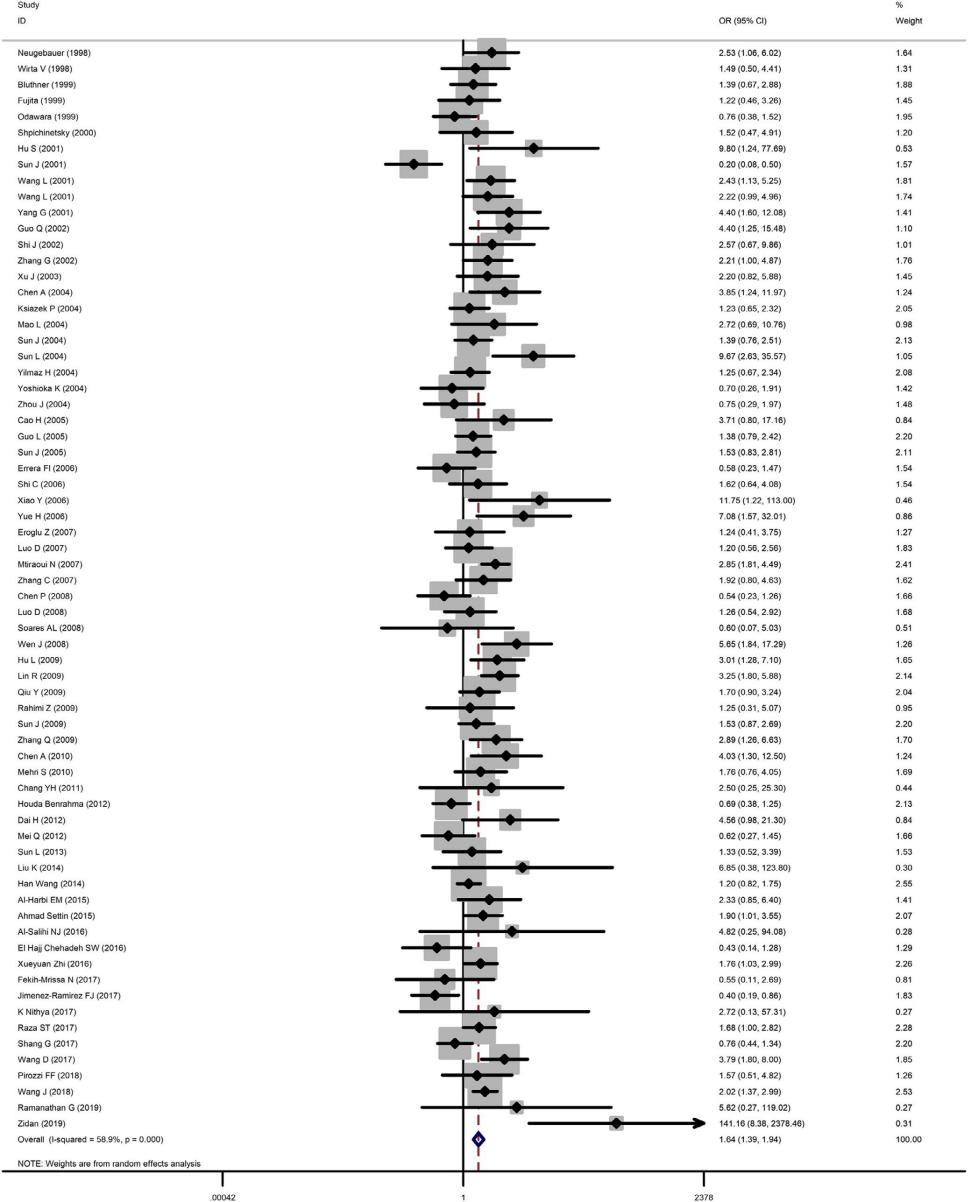
Forest plot of association between *MTHFR* C677T polymorphism and T2DM under homozygous model. The horizontal lines represent the study‐specific ORs and 95% CIs, respectively. The diamond represents the pooled results of OR and 95% CI. CI, confidence interval; OR, odds ratio; T2DM, type 2 diabetes mellitus

Table 2 and Figure 2 illustrated the main results of the current meta‐analysis. We adopted five genetic models to assess the association between *MTHFR* C677T and T2DM: homozygote model TT versus CC, heterozygous model CT versus CC, recessive model TT versus CT/CC, dominant model CT/TT versus CC, and allele model T versus C. There was a significant association between *MTHFR* C677T polymorphism and T2DM under homozygous (OR = 1.64, 95% CI = 1.39–1.94), heterozygous (OR = 1.38, 95% CI = 1.20–1.59), recessive (OR = 1.41, 95% CI = 1.23–1.61), dominant (OR = 1.47, 95% CI = 1.27–1.70), and allele (OR = 1.37, 95% CI = 1.23–1.52) genetic models in a random‐effects model.

**Table 2:** Meta‐analysis of the association between *MTHFR* C677T polymorphism and T2DM susceptibility

Variables	No. of studies	Homozygous		Heterozygous		Recessive		Dominant		Allele	
		TT versus CC		CT versus CC		TT versus CT/CC		CT/TT versus CC		T versus C	
		OR (95% CI)	*p^het^*	OR (95% CI)	*p^het^*	OR (95% CI)	*p^het^*	OR (95% CI)	*p^het^*	OR (95% CI)	*p^het^*
All	68	**1.64 (1.39–1.94)**	<.001	**1.38 (1.20–1.59)**	<.001	**1.41 (1.23–1.61)**	<0.001	**1.47 (1.27–1.70)**	<.001	**1.37 (1.23–1.52)**	<.001
Ethnicity											
Asian	52	**1.78 (1.48–2.15)**	<.001	**1.51 (1.33–1.70)**	<.001	**1.43 (1.23–1.67)**	<0.001	**1.60 (1.40–1.82)**	<.001	**1.44 (1.29–1.59)**	<.001
Caucasian	11	1.20 (0.81–1.79)	.007	0.79 (0.52–1.21)	<.001	**1.43 (1.14–1.79)**	0.457	0.87 (0.57–1.32)	<.001	0.97 (0.72–1.32)	<.001
African	5	1.70 (0.63–4.57)	<.001	1.88(0.75–4.74)	<.001	1.45 (0.62–3.39)	0.002	2.15 (0.86–5.42)	<.001	1.92 (0.98–3.73)	<.001
HWE											
>0.05	52	**1.76 (1.45–2.13)**	<.001	**1.34 (1.14–1.57)**	<.001	**1.50 (1.29–1.75)**	0.006	**1.48 (1.26–1.73)**	<.001	**1.39 (1.24–1.56)**	<.001
≤0.05	16	1.38 (0.99–1.92)	<.001	**1.48 (1.11–1.99)**	<.001	1.19 (0.92–1.55)	<0.001	**1.44 (1.07–1.95)**	<.001	**1.29 (1.01–1.65)**	<.001
Quality score											
>9	20	**1.46 (1.12–1.89)**	<.001	1.29 (0.99–1.69)	<.001	**1.37 (1.12–1.68)**	0.003	**1.36 (1.05–1.75)**	<.001	**1.30 (1.09–1.56)**	<.001
≤9	48	**1.78 (1.43–2.23)**	<.001	**1.42 (1.21–1.67)**	<.001	**1.45 (1.21–1.73)**	0.001	**1.53 (1.29–1.82)**	<.001	**1.40 (1.23–1.60)**	<.001

Note

The GenBank reference sequence and version number for the gene is: *MTHFR* (NM_005957.5). Values were in bold if 95% CIs excluded 1 or *p* values less than .05.

Abbreviations: CI, confidence interval; Het, heterogeneity; HWE, Hardy–Weinberg equilibrium; OR, odds ratio; T2DM, type 2 diabetes mellitus.

In the subgroup analysis based on ethnicity, we divided the included studies into three ethnicities: Asian, Caucasian, and African. We found significant association between *MTHFR* C677T genotype and T2DM in Asian populations, under each genetic models homozygous (OR = 1.78, 95% CI = 1.48–2.15), heterozygous (OR = 1.51, 95% CI = 1.33–1.70), recessive (OR = 1.43, 95% CI = 1.23–1.67), dominant (OR = 1.60, 95% CI = 1.40–1.82), and allele (OR = 1.44, 95% CI = 1.29–1.59). However, no relationship was found between *MTHFR* C677T genotype and T2DM in Caucasian and African, except for the recessive model in Caucasian group (OR = 1.43, 95% CI = 1.14–1.79). When stratified by HWE, significant association was also observed in the subgroup of HWE >0.05, under homozygous (OR = 1.76, 95% CI = 1.45–2.13), heterozygous (OR = 1.34, 95% CI = 1.14–1.57), recessive (OR = 1.50, 95% CI = 1.29–1.75), dominant (OR = 1.48, 95% CI = 1.26–1.73), and allele (OR = 1.39, 95% CI = 1.24–1.56) genetic models. Significant association was detected in heterozygous (OR = 1.48, 95% CI = 1.11–1.99), dominant (OR = 1.44, 95% CI = 1.07–1.95), and allele (OR = 1.29, 95% CI = 1.01–1.65) in the subgroup of HWE < 0.05. A subgroup analysis stratified by quality score was also conducted. Significant association was detected in homozygous (OR = 1.46, 95% CI = 1.12–1.89), recessive (OR = 1.37, 95% CI = 1.12–1.68), dominant (OR = 1.36, 95% CI = 1.05–1.75), and allele (OR = 1.30, 95% CI = 1.09–1.56) genetic models, in the subgroup of quality score > 9. Significant association was also detected in homozygous (OR = 1.78, 95% CI = 1.43–2.23), heterozygous (OR = 1.42, 95% CI = 1.21–1.67), recessive (OR = 1.45, 95% CI = 1.21–1.73), dominant (OR = 1.53, 95% CI = 1.29–1.82), and allele (OR = 1.40, 95% CI = 1.23–1.60) genetic models, in the subgroup of quality score ≤9.

### 3.4 Heterogeneity and sensitivity analysis

As shown in Table 1, substantial heterogeneities could be found among all the genetic models (*p* < .001) for the *MTHFR* C677T. Therefore, the random‐effect model was used to calculate the pooled ORs and 95% CIs for all the models.

**Figure 3:**
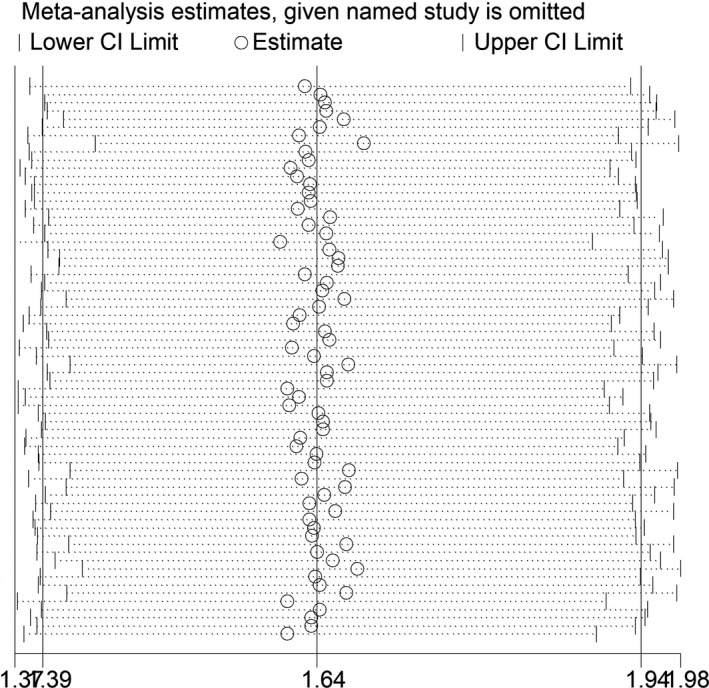
Sensitivity analysis of the association between *MTHFR* C677T polymorphism and T2DM. Each point represents the recalculated OR after deleting a separate study. OR, odds ratio; T2DM, type 2 diabetes mellitus

Sensitivity analysis using sequential leave‐one‐out strategy was carried out to explore the influence of a single study on the pooled ORs. The omission of each study did not impact the recalculated ORs, indicating the credibility and reliability of our results (Figure 3).

### 3.5 Publication bias

**Figure 4:**
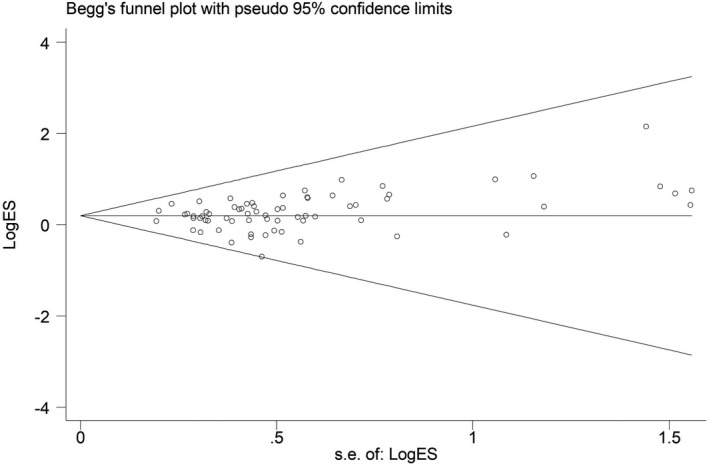
Funnel plot analysis for assessing publication bias for *MTHFR* C677T polymorphism under homozygous model. Each point represents a separate study for the indicated association

Begg's funnel plot and quantitative Egger's test were adopted to test the publication bias of the current meta‐analysis. As indicated by the symmetrical shape of the Begg's funnel plots, no significant publication bias was observed (Figure 4). Moreover, Egger's test also suggested the nonexistence of publication bias among the studies (data not shown).

## 4 DISCUSSION

To our knowledge, the current meta‐analysis represents the largest and most comprehensive one regarding the relationship between *MTHFR* C677T and T2DM so far. Our analysis provided strong evidence that *MTHFR* C677T was significantly associated with T2DM, especially in Asians. Sensitivity analysis indicated that there was no significant change in the overall results by removing one study in each turn. Publication bias analysis also showed that the results are convincible.

*MTHFR* C677T is a functional genetic variation that leads to amino acid substitution from alanine to valine (Ueland, Hustad, Schneede, Refsum, & Vollset, 2001). Such amino acid shift was illustrated to compromise the enzyme activity to nearly 50%, compared to the wild‐type MTHFR enzyme (Weisberg et al., 1998). Although the relationship between T2DM susceptibility and *MTHFR* C677T genotype has been largely investigated, contradictory conclusions still remain. In 2006, no evidence of association was found by F.I.V. Errera et al. between the 677TT genotype of *MTHFR* and T2DM, in Brazilian populations (Errera et al., 2006). In a study conducted in China in 2014, Wang et al. found that C677T in the *MTHFR* may influence the risk of T2DM (Wang et al., 2014). Recently, in a case–control study conducted in the population of Brazilian with 47 T2DM cases and 78 controls by Flavio Fontes Pirozzi et al. (Pirozzi et al., 2018), no correlation was found between the *MTHFR* C677T in the development of T2DM.

Due to the divergent results among single‐country studies, several systematic meta‐analyses have been undertaken to determine conclusively whether *MTHFR* C677T is associated with the risk of T2DM. In 2013, Chinese academics Zhong, Rodriguez, Yang, and Li (2013) conducted a meta‐analysis regarding *MTHFR* C677T and T2DM. Their meta‐analysis included 4,855 T2DM patients and 5,242 controls. However, they failed to obtain clear evidence of a significant association of *MTHFR* C677T and T2DM across all 39 studies conducted in 15 countries. They also failed to provide compelling evidence of an association specifically for African, Asian, or Caucasian populations. Interestingly, Khalid et al. (Al‐Rubeaan et al., 2013) observed that there was a significant relationship between *MTHFR* C677T polymorphism and T2DM in Arab population, in 2013. In 2014, Zhu et al. (2014) conducted an updated meta‐analysis in Chinese population aiming to better identify the role of C677T polymorphism in T2DM. They included 29 studies with 4,656 T2DM patients and 2,127 controls. They detected a significant relationship between *MTHFR* C677T polymorphism and T2DM in the Chinese Han population.

Genotype frequencies at the C677T locus of *MTHFR* vary widely by ethnicity (Errera et al., 2006; Yilmaz et al., 2004), raising the possibility that any association between this SNP and the risk of T2DM may likewise depend on ethnicity. Thus, we further put our focus on ethnic stratification analysis based on the groups that emerged from our literature searches: African, Asian, and Caucasian. Our analysis provided strong evidence that *MTHFR* C677T was significantly associated with T2DM in Asians, but not in Caucasians or Africans. Thus, it is necessary to identify the role of C677T in different ethnicities.

Several weaknesses should be pointed out before interpreting our conclusion. First, selection bias could not be avoided as only the articles in English and Chinese were analyzed. Other studies written in other languages were unable to include. Second, analyzing one SNP in *MTHFR* was far more enough, as the development of T2DM was associated with multiple SNPs in multiple genes. Third, we also failed to determine the role of other potential influential factors in the initiation of T2DM. These potential influential factors such as life style, environment exposures, and gene–environment interactions were reported to be associated with T2DM. Fourth, it is inevitable to avoid several shortages such as misclassified genotypes, unwell‐matched sources of controls, and inconsistent qualities of the included studies, due to the retrospective nature of meta‐analysis. Finally, between‐study heterogeneity was found in all comparisons, which may compromise the reliability of conclusion.

## 5 CONCLUSION

In all, this meta‐analysis provides a precise conclusion that *MTHFR* C677T polymorphism was significantly associated with T2DM, especially in Asian populations. Further well‐designed, large‐scale, and in‐depth studies are warranted to check such relationship.

## CONFLICT OF INTEREST

The authors declare that they have no conflict of interest.

## AUTHOR CONTRIBUTIONS

Y.M. and X.L. conceived the study design and wrote the paper. K.M., L.Z., M.L., and M.Z. performed the selection, collected the data, and performed the statistical analysis. M.G. and G.Q. were responsible for the quality control of data. All authors read and approved the final manuscript.
